# Adult T-Cell Leukemia/Lymphoma With Extranodal Involvement of the Hypopharynx

**DOI:** 10.7759/cureus.22723

**Published:** 2022-02-28

**Authors:** Shohei Fujimoto, Kazuto Matsuura, Hirotaka Nakamura, Shingo Sakashita, Ryuichi Hayashi

**Affiliations:** 1 Head and Neck Surgery, National Cancer Center Hospital East, Kashiwa, JPN; 2 Hematology, National Cancer Center Hospital East, Kashiwa, JPN; 3 Pathology and Laboratory Medicine, National Cancer Center Hospital East, Kashiwa, JPN

**Keywords:** flower cell, head and neck, hypopharynx, htlv-1, extranodal involvement, adult t-cell leukemia/lymphoma

## Abstract

Adult T-cell leukemia/lymphoma (ATLL) with extranodal lesions in the head and neck region is rare, with only a few cases reported so far. To our knowledge, this the first case reported involving the hypopharynx. The patient in this case was a 49-year-old woman who presented to an otolaryngology clinic complaining of an abnormal sensation in the throat and difficulty in swallowing. Endoscopic examination revealed an irregular mass with a smooth surface centered on the left piriform sinus and extending to the arytenoid and post-cricoid area. Imaging studies revealed lymphadenopathy in multiple cervical lymph nodes and in the hepatoduodenal mesentery. Examination of a peripheral blood smear revealed proliferation of atypical lymphocytes including flower cells. Further history-taking revealed that both the patient and her parents were positive for human T-cell lymphotropic virus type 1 (HTLV-1) antibodies and that her sister had developed ATLL. Lymph node biopsy was performed; hematoxylin-eosin staining of it showed a slight increase in atypical large lymphocytes, and flow cytometry of it showed CD4 and CD25 positivity in large lymphocytes. In peripheral blood samples, Southern blotting detected the monoclonal integration of the HTLV-1 provirus. Based on these findings, we diagnosed the patient with ATLL with extranodal involvement. ATLL should be included in the differential diagnosis for malignant lymphoma of the head and neck. A detailed medical interview and cooperation with the pathological department are key to a prompt diagnosis.

## Introduction

Adult T-cell leukemia/lymphoma (ATLL) is associated with human T-cell lymphotropic virus type 1 (HTLV-1) infection and is characterized by neoplastic proliferation of CD4-positive T-cells. Although it is known that extranodal lesions occur in various organs in ATLL patients, including the skin, intestinal tract, and central nervous system, extranodal involvement of the head and neck is uncommon [1]. There are a few reports of pharyngeal lesions around Waldeyer’s ring, but, to our knowledge, no reports of the hypopharynx in the Japanese and English literature. Here, we report the first case of acute-type ATLL that presented initially as a hypopharyngeal lesion.

## Case presentation

A 49-year-old woman, from Kyushu, Japan, with no known past medical history presented to an otolaryngology clinic with a two-week history of an abnormal sensation in the throat and difficulty in swallowing. She was a never-smoker and a social drinker. Both the patient and her parents were positive for HTLV-1 antibodies; her sister had developed ATLL two years earlier and undergone hematopoietic stem cell transplantation. The patient had no history of blood transfusion. Clinical examination revealed a tumor in the left hypopharyngeal region, so she was referred to our hospital for further examination and treatment.

**Figure 1:**
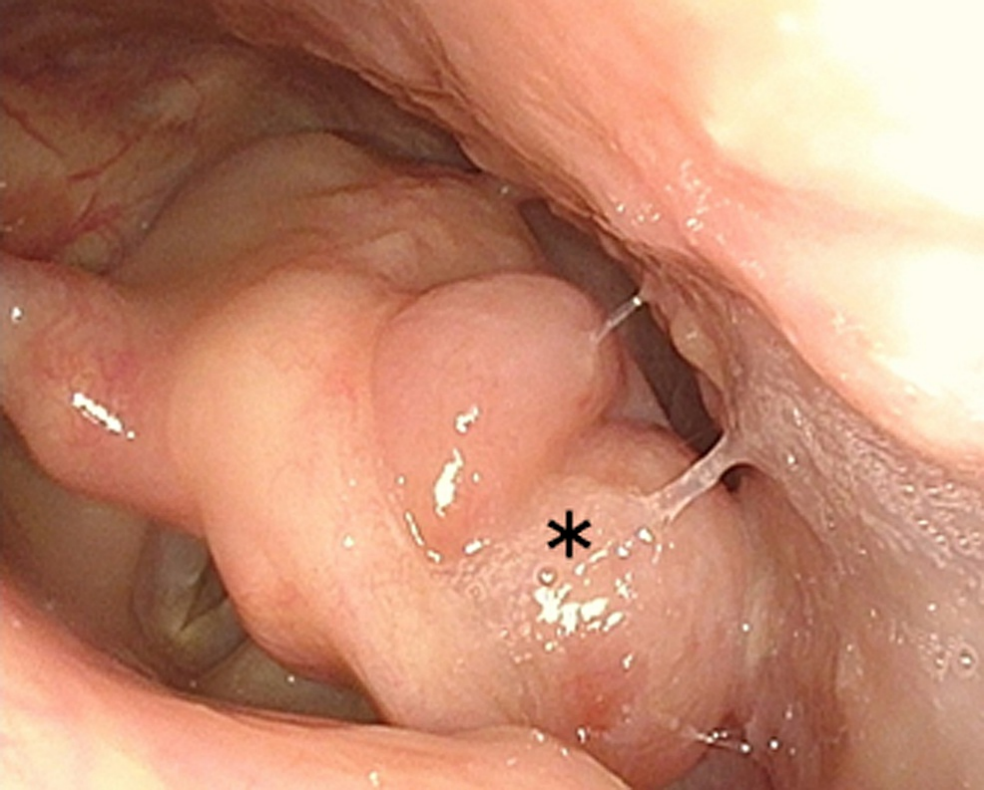
Endoscopic image of the pharynx showing an irregular mass with a smooth surface centered on the left piriform sinus and extending to the arytenoid and post-cricoid area (shown by *)

Endoscopic examination revealed an irregular mass with a smooth surface centered on the left piriform sinus and extending to the arytenoid and post-cricoid area (Figure 1). On narrow-band imaging, there were no brownish areas around the tumor indicating mucosal extension of the tumor. No other abnormalities were found in the hypopharyngeal or laryngeal region. There was no vocal cord paralysis.

**Figure 2:**
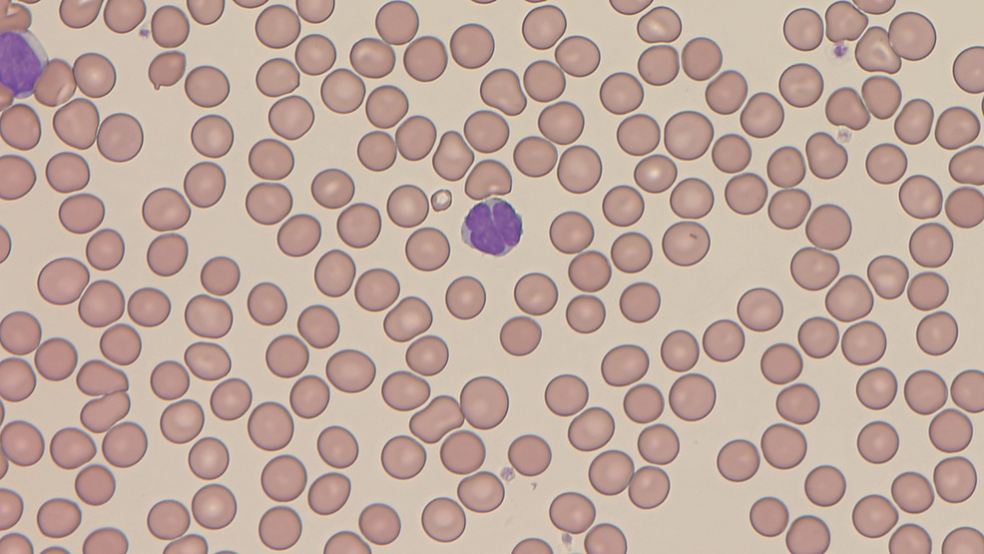
Findings of a peripheral blood smear (×600) Atypical lymphocytes including flower cells (center of the figure) that have highly indented or lobulated nuclei with condensed chromatin were observed in peripheral blood. Flower cells are characteristic of adult T-cell leukemia/lymphoma and observed much in the acute type.

Examination of blood chemistry and a peripheral blood smear revealed the following: white blood cell count 10,100/µL, lymphocytes 47.9%, atypical lymphocytes 17.0% (including flower cells that have highly indented or lobulated nuclei with condensed chromatin), red blood cells 512×10^4^/µL, platelets 25.5×10^4^/µL, albumin 4.8 g/dL, blood urea nitrogen 7 mg/dL, calcium 9.9 mg/dL, lactate dehydrogenase (LDH) 162 IU/L, an interleukin-2 receptor level of 3450 U/mL, and an HTLV-1 antibody level of ≥256 (Figure 2).

**Figure 3:**
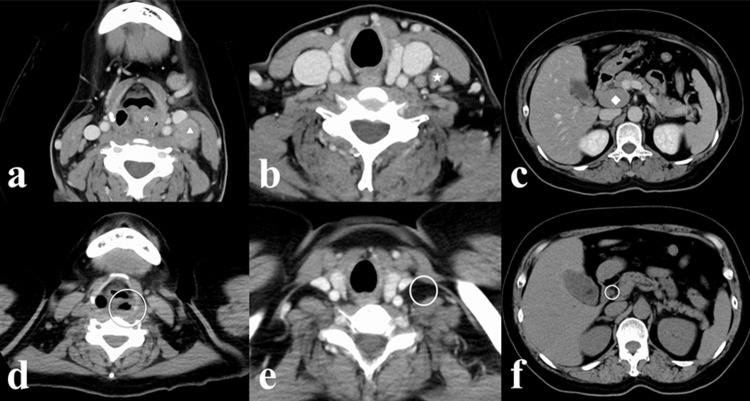
Findings on computed tomography (a-c) A contrast-enhanced scan obtained on initial presentation shows a 15×11×22 mm iso-density mass centered on the left piriform sinus extending to the arytenoid and post-cricoid area and bulged toward the laryngeal cavity (*). Lymphadenopathy was observed in the left superior (△), middle (☆), and inferior internal jugular nodes of the cervical region and in the hepatoduodenal mesentery (◇). (d-f) After one course of chemotherapy using the mLSG15 protocol, there was a marked reduction in the size of the lesion.

Contrast-enhanced computed tomography showed a 15×11×22 mm iso-density mass centered on the left piriform sinus extending to the arytenoid and post-cricoid area and bulged toward the laryngeal cavity. There was lymphadenopathy in the left superior, middle, and inferior internal jugular nodes in the cervical region and in the hepatoduodenal mesentery (Figures 3a-3c). Magnetic resonance imaging showed the tumor lesion to have relatively well-defined borders and low signal intensity on T1-weighted images, uniformly medium to high signal intensity on T2-weighted images, homogenous internal signal, and a low apparent diffusion coefficient (0.57×10^-3 ^mm^2^/sec). There was no extralaryngeal extension or cartilage invasion.

**Figure 4:**
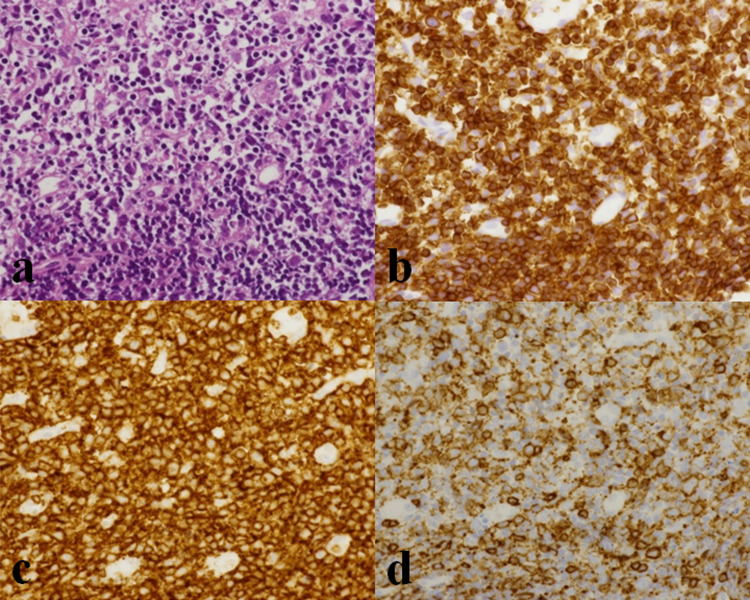
Histological and immunohistochemical studies (a) Hematoxylin-eosin staining of a hypopharyngeal lesion biopsy specimen showed diffuse proliferation of small- to medium-sized lymphocytes (×400). Immunostaining of a cervical lymph node biopsy specimen showed (b) CD3 positivity, and a bias toward (c) CD4 > (d) CD8 (×400).

The clinical findings of a hypopharyngeal lesion suggested lymphoma and cancer as differential diagnoses. On the basis of her family history, we considered the possibility of ATLL with extranodal involvement. A biopsy of the hypopharyngeal tumor suggested T-cell lymphoma. Hematoxylin-eosin staining of it showed diffuse proliferation of small- to medium-sized lymphocytes. Immunostaining of it showed a bias toward CD3 > CD20, CD3 > CD5, and CD4 > CD8 (Figure 4). A cervical lymph node biopsy was performed to obtain a larger sample volume. Hematoxylin-eosin staining of it showed a slight increase in atypical large lymphocytes. Flow cytometry of it showed a bias toward CD4 > CD8, CD25 positivity, and decreased expression of CD7 in large lymphocytes. In peripheral blood samples, Southern blotting detected monoclonal integration of HTLV-1 provirus. Based on these findings, we diagnosed the patient with acute-type ATLL with extranodal involvement.

The patient was referred to the hematology department, where chemotherapy using the mLSG15 protocol, which comprises VCAP (vincristine, cyclophosphamide, doxorubicin, and prednisone), AMP (doxorubicin, ranimustine, and prednisone), and VECP (vindesine, etoposide, carboplatin, and prednisone), was performed, and the size of the hypopharyngeal, nodal and hepatoduodenal mesentery lesions was found to be markedly reduced (Figures 3d-3f). After three courses of mLSG15, the lesions showed complete response although atypical lymphocytes were still present in the peripheral blood. The patient underwent HLA-haploidentical stem cell transplantation with her daughter as the donor. Thereafter, the clinical course was favorable with no major adverse events, and the atypical lymphocytes in the peripheral blood disappeared, resulting in complete remission. As of this writing, the patient has maintained complete remission for exactly one year after transplantation. Left vocal cord paralysis was observed after the cervical lymph node biopsy, and the patient remains under observation in our department.

## Discussion

According to the 2017 World Health Organization classification, ATLL is classified independently from other hematopoietic tumors as a peripheral T-cell tumor caused by HTLV-1 infection, and consists of lymphocytes with a high degree of nuclear atypia [1]. The disease causes leukocytosis (mainly due to the proliferation of atypical lymphocytes including flower cells), lymphadenopathy, skin lesions, multiple organ damage due to infiltration of ATLL cells, a high serum LDH level, hypercalcemia, and opportunistic infections [2]. The highest prevalence of ATLL is found on the southwest part of Japan, Central Africa, and Latin America. There are about 1.1 million HTLV-1 carriers, mainly in the Kyushu and Okinawa regions in Japan, and the annual incidence of ATLL among HTLV-1 carriers is 0.6-0.7 per 1000 population [3,4]. Blood transfusions, sexual intercourse, and breast milk are known routes of transmission.

Diagnosis of ATLL requires genetic evidence of the monoclonal integration of HTLV-1 provirus by Southern blotting or polymerase chain reaction. Most cases show CD4, CD25, and CCR4 positivity and have the same immunophenotype as regulatory T-cells. CD7 expression is often lost [5]. Based on clinical and blood biochemistry findings, ATLL is classified as acute type (57%), lymphoma type (19%), chronic type (19%), or smoldering type (6%). The acute type and lymphoma type are considered high-grade ATLL and often have a rapid course, with a median survival of 6.2 and 10.2 months, respectively [6]. The diagnostic criteria for each type are shown in Table 1. In the present case, diagnosis of the acute type was based on the discovery of flower cells in peripheral blood, and other diagnostic criteria for lymphoma type, chronic type, or smoldering type were not met. Chemotherapy, such as the mLSG15 protocol, is recommended for high-grade ATLL. However, given that ATLL cells gradually become chemoresistant, hematopoietic stem cell transplantation is generally performed after chemotherapy to reduce the tumor volume if a complete cure is sought. If the patient is too old or otherwise ineligible for hematopoietic stem cell transplantation, novel therapies, such as mogamulizumab and lenalidomide, may be considered. However, it is difficult to control the disease in the long term using these therapies.

**Table 1:** Diagnostic criteria and clinical subtypes of ATLL ATLL, adult T-cell leukemia/lymphoma; HTLV-1, human T-cell lymphotropic virus type 1; N, upper limit of normal Diagnostic criteria and clinical subtypes of ATLL were modified from reference [7].

Assessment item		Smoldering type	Chronic type	Lymphoma type	Acute type
HTLV-1 provirus		+	+	+	+
Lymphocytes (per mm^3^)		<4000	≥4000	<4000	Not specified
Atypical lymphocytes		≥5%	+	≤1%	+
Flower cells		Sometimes	Sometimes	-	+
Lactate dehydrogenase		≤1.5 N	≤2.0 N	Not specified	Not specified
Corrected calcium level (mg/dL)		<11.0	<11.0	Not specified	Not specified
Lymphadenopathy (histologically confirmed tumor lesions)		-	Not specified	+	Not specified
Localization of the tumor lesion	Skin	Not specified	Not specified	Not specified	Not specified
	Lung	Not specified	Not specified	Not specified	Not specified
	Lymph node	-	Not specified	+	Not specified
	Hepatomegaly	-	Not specified	Not specified	Not specified
	Central nerves	-	-	Not specified	Not specified
	Bone	-	-	Not specified	Not specified
	Pleural effusion	-	-	Not specified	Not specified
	Ascites	-	-	Not specified	Not specified
	Gastrointestinal tract	-	-	Not specified	Not specified

In ATLL, extranodal tumors of the head and neck are rare. A report by the Lymphoma Study Group describes tumors in the skin, lungs, lymph nodes, central nervous system bone, and gastrointestinal tract, hepatomegaly, splenomegaly, pleural effusion, and ascites, but not extranodal lesions of the head and neck [6]. Miyagi et al. reported nine cases of ATLL with extranodal lesions of the head and neck and identified a total of 40 cases in the literature. Lesions were of the nasal sinonasal tract, salivary gland, Waldeyer's ring, oral cavity, larynx, pharynx (hypopharynx was not included), and others, in that order. They reported that patients with localized extranodal lesions of the head and neck had a longer median survival than those with systemic lesions (39.6 vs. 8.1 months) [7]. Kojya et al. reported 10 cases of lymphoma-type ATLL involving Waldeyer's ring, in Okinawa. Primary treatment was successful in many cases, but most of them had recurrences and had a poor prognosis. They mentioned the importance of early detection in localized extranodal lesions of the head and neck [8]. To our knowledge, this study reports the first case of ATLL of the hypopharynx. Further accumulation of cases is needed to clarify the characteristics and prognosis of ATLL in the head and neck region.

ATLL is characterized by regional and vertical transmission, and it is important to obtain a detailed history to make a diagnosis. In the present case, ATLL was suspected because the patient was from Kyushu; both the patient and her parents had HTLV-1 antibodies, and her older sister had developed ATLL. In areas where HTLV-1 carriers are common, patients with head and neck cancer are screened for HTLV-1, along with hepatitis B and hepatitis C virus [9]. Malignant lymphomas, including ATLL, have been classified into a wide variety of pathological types, and the diagnosis often requires a comprehensive approach that includes morphological examination under the microscope as well as immunostaining, flow cytometry, and chromosomal and genetic analysis. Therefore, it is important for clinicians to consult with pathologists in order to promptly diagnose this disease and initiate treatment.

Finally, we discuss the cause of the vocal cord paralysis. We do not believe that the lymph node biopsy was the cause, because it was performed in the left middle internal jugular region (Figure 3b) and thus would not affect the vagus or recurrent nerve. In rare cases, ATLL may infiltrate the central peripheral nerves or cranial nerves (neurolymphomatosis), but in this case, there was no progression of neurological symptoms other than hoarseness during the clinical course. Also, no abnormal accumulation in the nerve was seen on positron emission tomography-computed tomography scans obtained to determine the effect of chemotherapy. We hypothesized that the tumor in the hypopharynx might have directly invaded the adjacent recurrent laryngeal nerve, but there has been no report of this to date.

## Conclusions

We have encountered a case of acute-type ATLL that initially presented as a hypopharyngeal lesion. After chemotherapy using the mLSG15 protocol, the patient underwent hematopoietic stem cell transplantation. Patients with malignant lymphoma often present first to an otolaryngology department or a head and neck department. ATLL should be included in the differential diagnosis for malignant lymphoma of the head and neck and can be confirmed promptly by a detailed medical interview and pathology consultation.
